# Clinical prognostic significance and pro-metastatic activity of RANK/RANKL via the AKT pathway in endometrial cancer

**DOI:** 10.18632/oncotarget.6795

**Published:** 2015-12-29

**Authors:** Jing Wang, Yao Liu, Lihua Wang, Xiao Sun, Yudong Wang

**Affiliations:** ^1^ Department of Gynecology, International Peace Maternity and Child Health Hospital, School of Medicine, Shanghai Jiao Tong University, Shanghai, China; ^2^ Laboratory for Gynecologic Oncology, International Peace Maternity and Child Health Hospital, School of Medicine, Shanghai Jiao Tong University, Shanghai, China

**Keywords:** RANK, RANKL, endometrial cancer, prognosis, metastasis

## Abstract

RANK/RANKL plays a key role in metastasis of certain malignant tumors, which makes it a promising target for developing novel therapeutic strategies for cancer. However, the prognostic value and pro-metastatic activity of RANK in endometrial cancer (EC) remain to be determined. Thus, the present study investigated the effect of RANK on the prognosis of EC patients, as well as the pro-metastatic activity of EC cells. The results indicated that those with high expression of RANK showed decreased overall survival and progression-free survival. Statistical analysis revealed the positive correlations between RANK/RANKL expression and metastasis-related factors. Additionally, RANK/RANKL significantly promoted cell migration/invasion via activating AKT/β-catenin/Snail pathway *in vitro*. However, RANK/RANKL-induced AKT activation could be suppressed after osteoprotegerin (OPG) treatment. Furthermore, the combination of medroxyprogesterone acetate (MPA) and RANKL could in turn attenuate the effect of RANKL alone. Similarly, MPA could partially inhibit the RANK-induced metastasis in an orthotopic mouse model via suppressing AKT/β-catenin/Snail pathway. Therefore, therapeutic inhibition of MPA in RANK/RANKL-induced metastasis was mediated by AKT/β-catenin/Snail pathway both *in vitro* and *in vivo*, suggesting a potential target of RANK for gene-based therapy for EC.

## INTRODUCTION

Endometrial cancer (EC) is the fourth most common cancer among women in the United States, with an estimated 54, 870 new cases and 10, 170 deaths in 2015 [1]. Besides well-known prognostic clinicopathological factors for survival in EC, such as histologic grade, clinical stage, and depth of myometrial invasion, tumor dissemination and metastasis have also been associated with survival [2]. Metastasis, the leading cause of death in EC, is initiated by compact regulation of associated molecules. It depends on the balance between pro-metastatic and anti-metastatic factors [3, 4].

Receptor activator of NF-κB (RANK) is expressed on several human prostate and breast cancer cell lines [5–7]. Receptor activator of NF-κB ligand (RANKL), the ligand of RANK, plays a pivotal, lineage-specific role in tumorigenesis and/or metastasis [8]. Binding to RANK, RANKL triggers a cascade of intracellular signaling events resulting in tumor metastasis [9–11]. Recently, evidences demonstrated that RANKL inhibition could contribute to a better clinical outcome [12]. Despite these encouraging data, the relationship between clinical outcome and tissue-based protein expression, especially RANK, is unclear.

Epidemiologic investigations have shown that long-term use of progestin is correlated with significant EC risk decrease [13–15]. However, women's health initiative (WHI) demonstrated that progestin increased the risk of several developing invasive cancers, including breast cancer, ovarian cancer and cardiovascular disease [16–18]. Dougall and Penninger demonstrated MPA could induce massive RANKL expression in the mammary gland via progestin receptor [19, 20]. RANKL, binding to RANK on mammary epithelial cells, promotes cell-cycle progression and protects these cells from apoptosis in response to DNA damage. These effects seem to be mediated by means of RANK/RANKL and IKK-α-NF-κB signaling [19, 20].

Metastasis is the primary reason for recurrence and death in EC patients, with a median survival of 7–12 months [21]. Our previous studies have demonstrated that RANK/RANKL is overexpressed in EC tissue specimens [22]. Considering the important role of RANK/RANKL in other tumors, the cause of tumor dissemination and metastasis in EC, particularly whether or how these processes are influenced by RANK/RANKL is needed further study.

Additionally, hormonal therapy with progestin has been identified as a favored therapeutic option to patients who have early-stage EC and desire future fertility with a response rate as high as 60% [23, 24]. Given the progestin-induced RANK/RANKL contributes to breast tumorigenesis, it is necessary to investigate the interaction between progestin and RANK/RANKL in EC. Thus, the point is whether and, if so, how RANK/RANKL contributes to the development and progression of EC, especially in the context of using MPA.

In the present study, we sought to evaluate the correlation between RANK expression and clinical outcome in EC. Meanwhile, we established an orthotopic xenograft EC model in nude mice to investigate the therapeutic efficacy of MPA alone and in combination with RANKL. We expect to demonstrate the stimulative role of RANKL in EC metastases and find the inhibition effect of MPA on RANK/RANKL. This will shed light on the mechanisms by which EC becomes more invasive and metastatic characteristics and provides a potential therapeutic target for EC.

## RESULTS

### Association of RANK expression with clinical outcome

**Figure 1:**
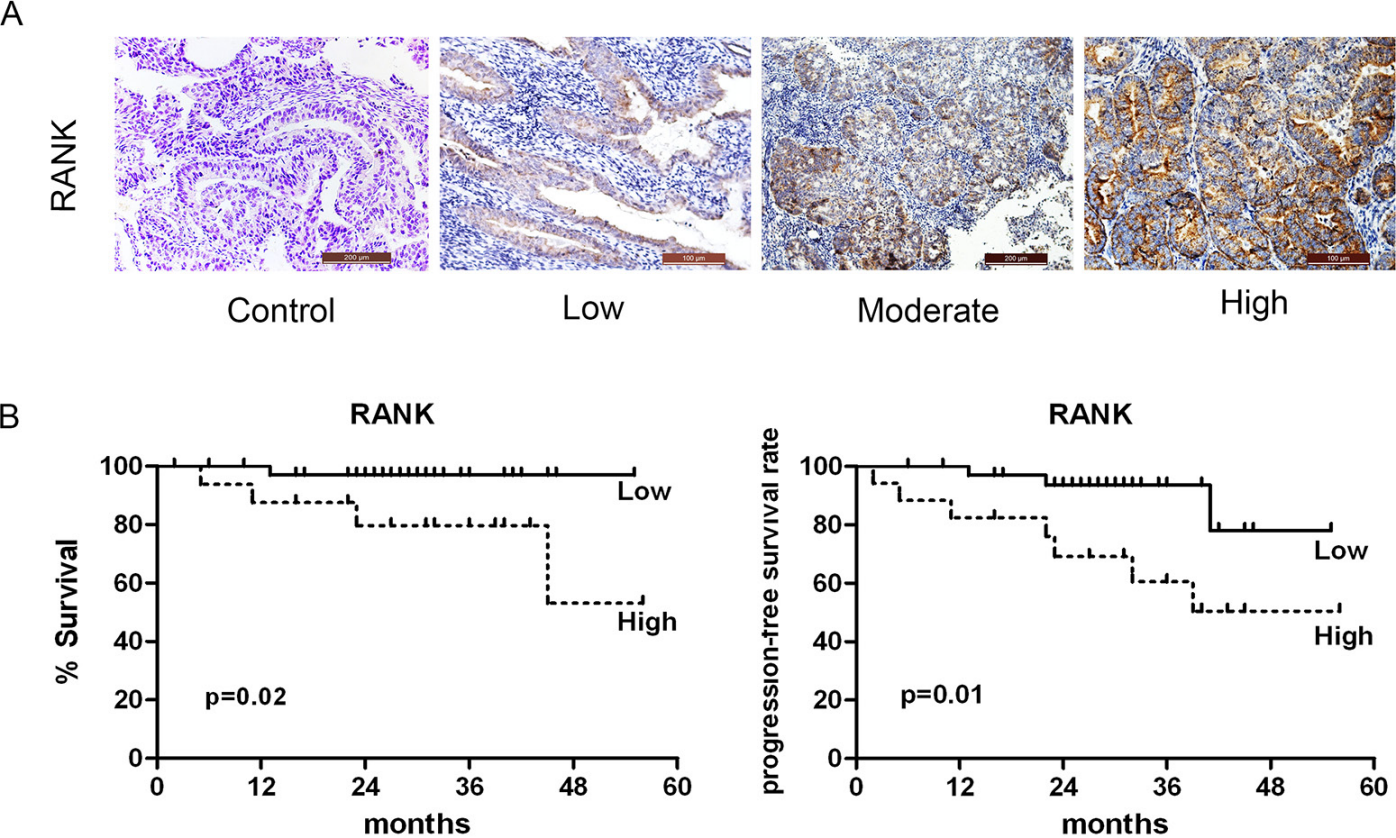
Association of RANK expression with clinical outcome (**A**) Immunohistochemical expression pattern of RANK in human endometrial adenocarcinoma specimens. (**B**) Kaplan-Meier survival curves for RANK expression. High level of RANK was significantly associated with decreased DSS.

Considering the relative scarcity of information about the clinical implication of RANK expression in human EC, we performed the first examination of 70 samples stained for RANK expression (Figure 1A). To assess the prognostic relevance of RANK, we did univariate survival analyses of traditional clinical parameters for disease-specific survival (DSS). Compared to those with low expression, patients with tumors exhibiting high level of RANK expression (*P* = 0.01) had a ∼ 5-fold higher risk of death. Furthermore, myometrial infiltration more than 50% (*P* = 0.03), lymph node involvement (*P* < 0.001) and vascular invasion (*P* = 0.01) had a 4-, 5- to 16-fold increased risk for death due to disease (Table 1). The Kaplan-Meier survival curve for RANK was shown in Figure 1B. To further investigate whether there was an independent correlation between any of the clinicopathologic parameters and DSS, we performed a multivariate Cox proportional hazards model. However, it failed to show a clear association between RANK expression and any of the following: age, grade, FIGO, histological type, or hormone receptor.

**Table 1:** Univariate survival analysis of prognostic variables for DSS

Variable	Median survival (month)	HR (95% CI)	*P*
RANK			
Low	NR		
High	NR	5.04 (1.35–18.83)	.01
Myometrial invasion			
< 1/2	NR		
≥ 1/2	56	4.00 (1.15–13.91)	.03
Positive lymph			
No	NR		
Yes	NR	16.41 (3.62–74.38)	< .001
Lymphovascular space involvement			
No	NR		
Yes	NR	5.12 (1.41–18.59)	.01

Abbreviations: CI, confidence interval; HR, hazard ratio; NR, not reached.

### Analysis of the Ishikawa-Luc/Ishikawa-Luc-Rank clones *in vitro*

**Figure 2:**
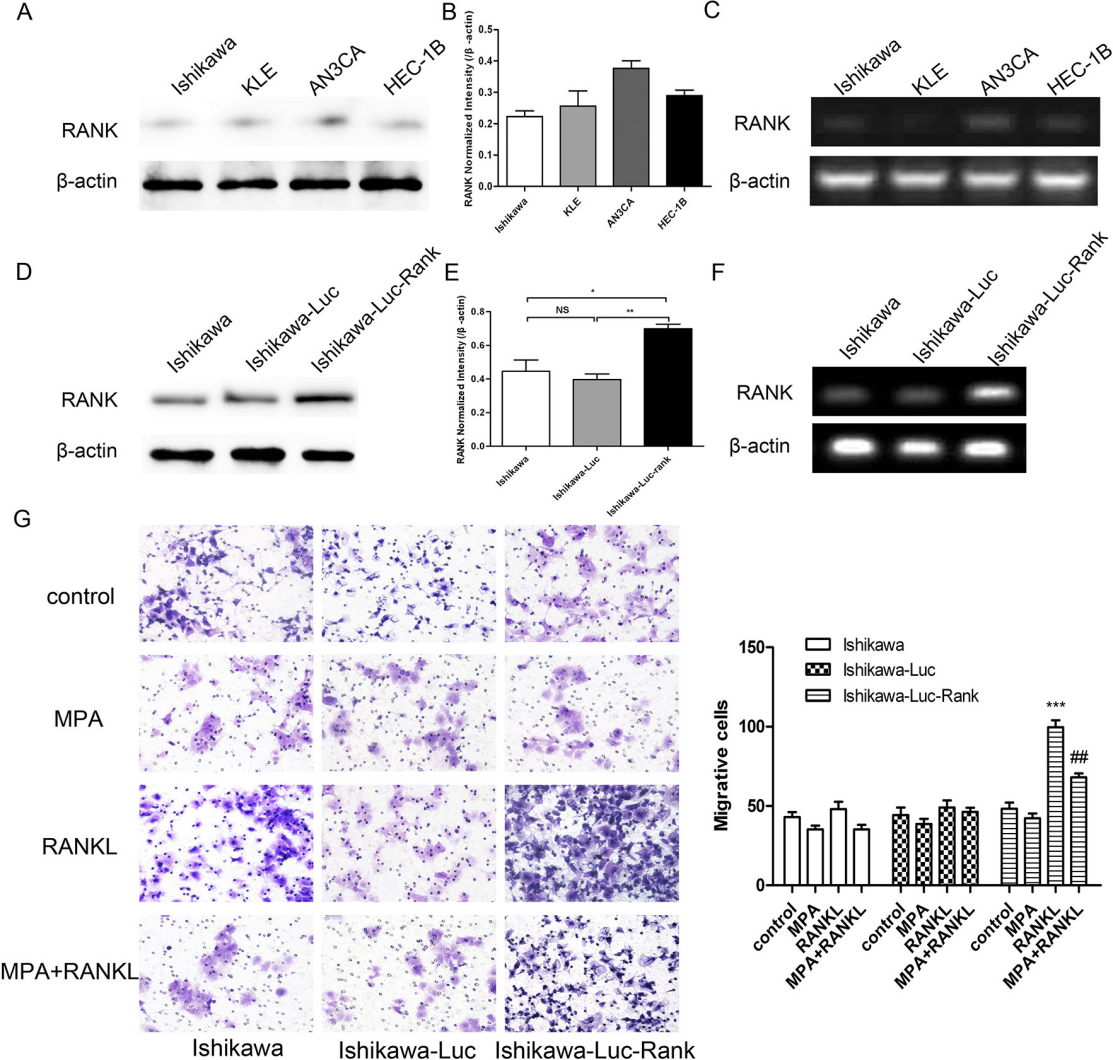
RANK promotes migration, invasion of EC cells (**A**) Determination of RANK expression in four EC cell lines. (**B**) Quantified by densitometry of triplicate blots. (**C**) RANK expression was analyzed by PCR. (**D**) Western blot analysis employed to validate the overexpression of the RANK. (**E**) Quantified by densitometry of triplicate blots. (**F**) PCR was also employed to validate the overexpression of the RANK gene. (**G**) RANKL promoted the migration of Ishikawa-Luc-Rank cell. Data were shown as the mean ± SEM (*P < 0.05,**P < 0.01,***P < 0.001 *vs* control; **P < 0.01 *vs* MPA-treat group).

To obtain Ishikawa-Luc/Ishikawa-Luc-Rank light-emitting tumor cells for the establishment of orthotopic xenograft model, human tumor cell line (Ishikawa) was transfected with the full-length luciferase gene. Furthermore, to determine the role of RANK/RANKL in EC progression, we stably transfected EC cell lines with a lentiviral vector expressing human RANK. Cells were transfected with an empty vector, which served as the control. These cell lines were named Ishikawa-Luc, and Ishikawa-Luc-Rank. Efficient transfection was confirmed before cellular assays were performed (Figure 2A–2F). Transwell migration and invasion assays demonstrated that the migratory and invasive capabilities of Ishikawa cells were significantly increased by RANK/RANKL, whereas MPA could suppress, at least in part, the migration and invasion induced by RANK/RANKL (Figure 2G).

### Activation of AKT/β-catenin/Snail pathway is involved in upregulation of RANK in Ishikawa cells

**Figure 3:**
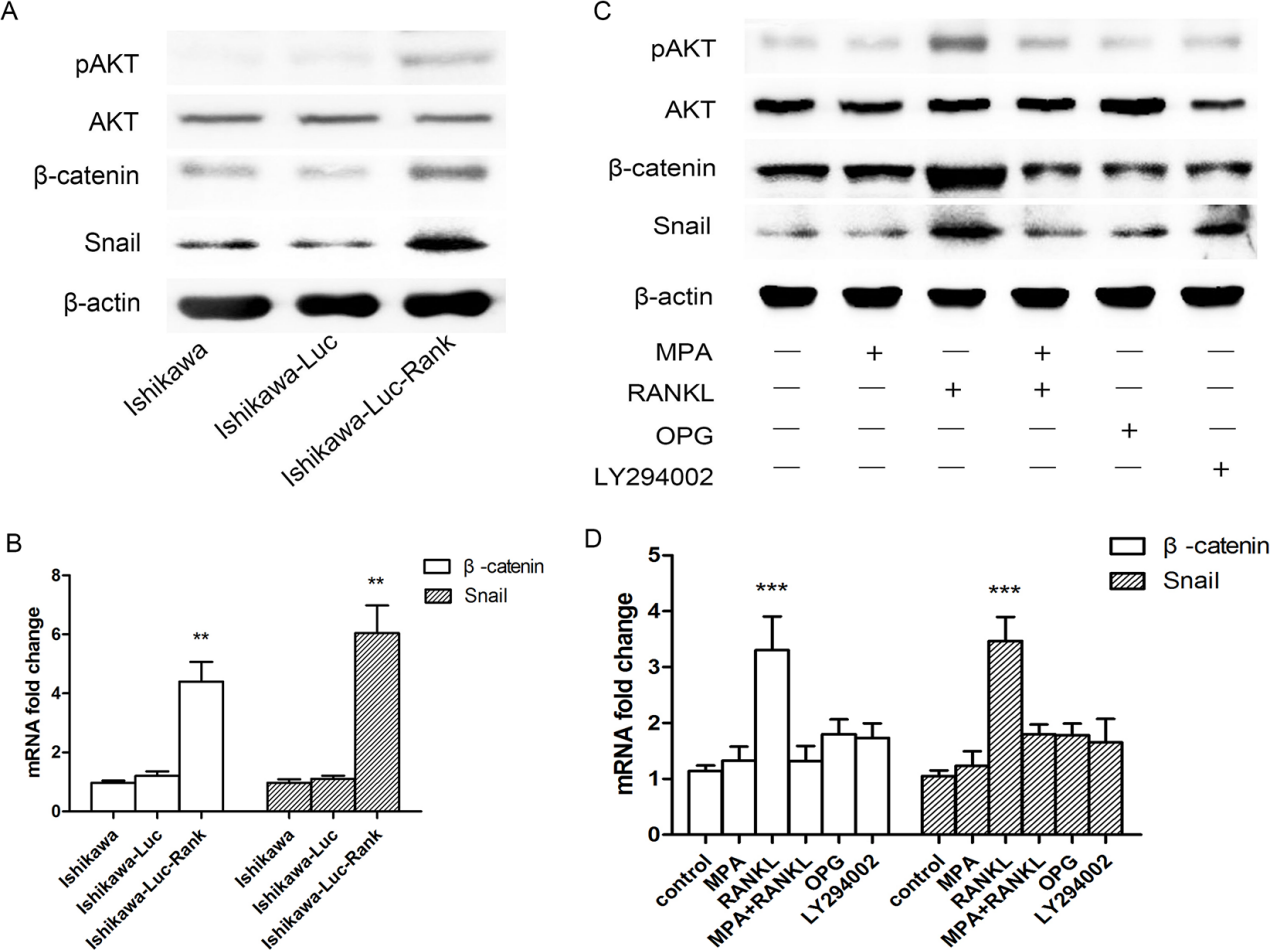
Activation of AKT/β-catenin/Snail pathway is involved in Ishikawa-Luc-Rank cells (**A**) The protein expression of pAKT, AKT, β-catenin and Snail of Ishikawa, Ishikawa-Luc, Ishikawa-Luc-Rank cells were examined by western blotting. (**B**) The mRNA expression of β-catenin and Snail of Ishikawa, Ishikawa-Luc, Ishikawa-Luc-Rank cells were examined by RT-PCR. (**C**) Ishikawa-Luc-Rank cells were treated with MPA, RANKL, OPG or LY294002 (10 μM) for 24 h, the pAKT, AKT, β-catenin and Snail were detected by western blotting. (**D**) Ishikawa-Luc-Rank cells were treated with MPA, RANKL, OPG or LY294002 (10 μM) for 24 h, the mRNA expression of β-catenin and Snail were detected by RT-PCR. Data were shown as the mean ± SEM (**P < 0.01, ***P < 0.001).

The AKT pathway is commonly activated in various cancers and plays a crucial role in promoting distant metastasis. To determine whether the AKT pathway is involved in tumor dissemination in Ishikawa-Luc-Rank cells, we examined the expression levels of pAKT and AKT by western blotting. The results showed that Ishikawa-Luc-Rank cells display an increased activation of AKT pathway compared to Ishikawa cells (Figure 3A). Given the AKT pathway strongly regulate β-catenin expression and activity, we therefore assessed whether the expression of β-catenin and its downstream gene Snail are enhanced in Ishikawa-Luc-Rank cells or not. Indeed, β-catenin level is substantially upregulated by RANK overexpression, as was Snail (Figure 3A). The expression of β-catenin and Snail at mRNA level was also increasing (Figure 3B). These results suggested that AKT/β-catenin/Snail signaling might mediate the effect of RANK on promoting metastasis.

To test the critical role of RANK/RANKL in regulation of AKT/β-catenin/Snail signaling in Ishikawa-Luc-Rank cells, a decoy receptor of RANKL, OPG was used. Additionally, to determine whether AKT signaling regulates β-catenin and Snail, a specific antagonist of PI3K/AKT, LY294002 was also used. After treating Ishikawa-Luc-Rank cells with MPA, RANKL, OPG, LY294002 and the combination of MPA and RANKL for 24 h, the expression of pAKT, AKT, β-catenin and Snail were determined. The results showed that blocking the binding of RANK and RANKL dramatically suppresses the expression of pAKT, β-catenin and Snail. Moreover, inactivation of AKT pathway significantly decreases the expression of β-catenin and Snail (Figure 3C). Suppression of AKT resulted in a distinct reduce of β-catenin and Snail expression at mRNA level (Figure 3D).

### Orthotopic tumor implantation generated a clinically relevant endometrial cancer model

The generation of an orthotopic xenograft model that recapitulates the pattern of EC development and metastasis would be useful for the further mechanism study. Primary tumors were derived from the injection of 1 × 10^7^ Ishikawa-Luc/Ishikawa-Luc-Rank cells in the flank of nude mice. The tumor formation time was approximately 14 days, and as can be observed macroscopically, the tumors were confined to subcutis. To have a model closer to what happens in clinical contexts, we implanted one tissue block which obtained from subcutaneous masses into the uterine cavity making a pocket (Supplementary Figure S1).

We speculated that the distant dissemination of tumor in this murine model arose through the same process that human EC cells underwent to infiltrate and metastasize. To certify the above assumption, mice were euthanized and *ex vivo* bioluminescence imaging (BLI) assay was performed at the end of the experiment. The organs were subsequently formalin-fixed and processed for histological examination by hematoxylin/eosin (H & E) staining. The endometrial generation of the tumor and the myometrial infiltration were confirmed by H & E staining. And the corresponding necropsy images showed the macroscopic metastasis (Supplementary Figure S2).

### RANK/RANKL results in orthotopic cancer development and distant micrometastasis generation

**Figure 4:**
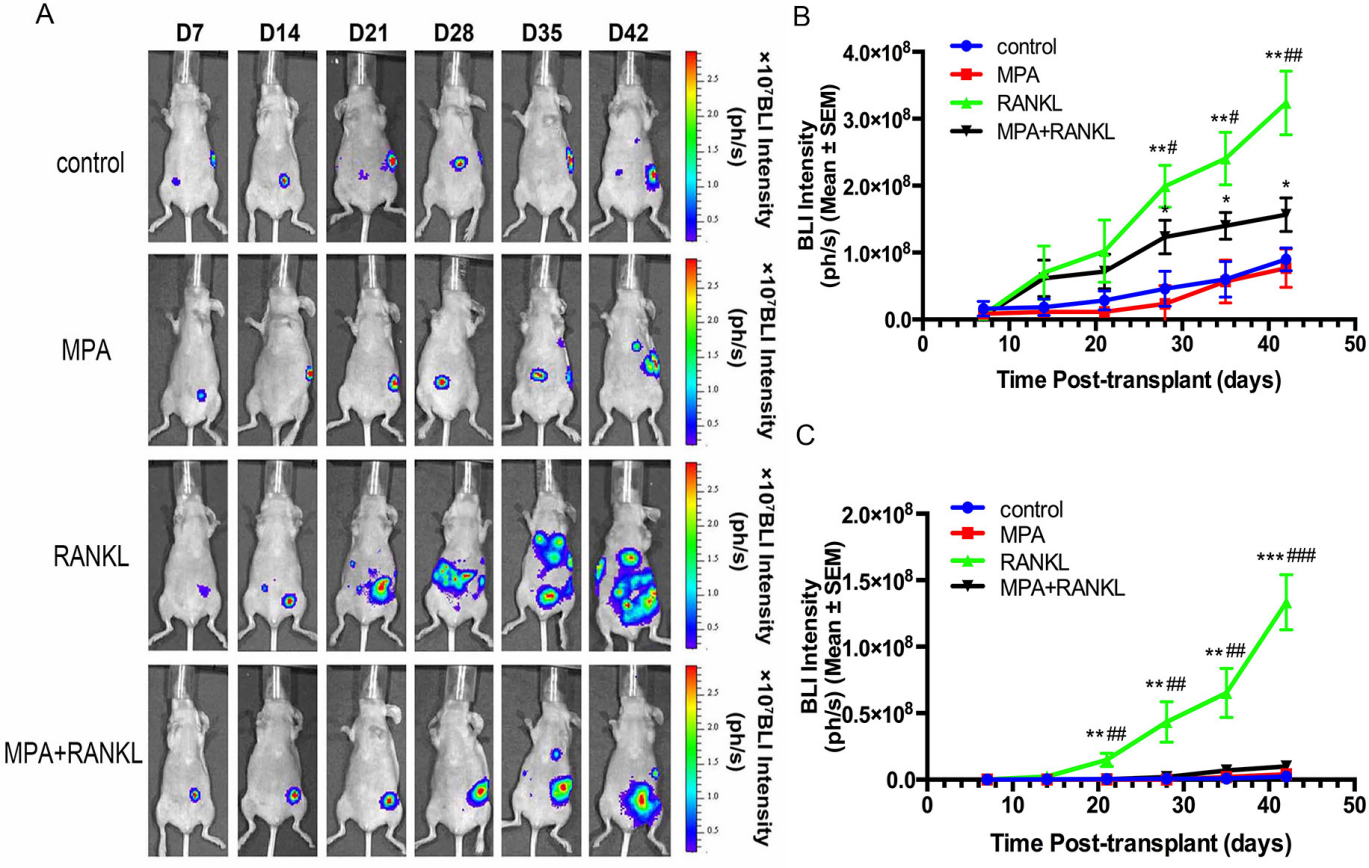
*In vivo* BLI of the orthotopic Ishikawa murine model Images were taken once a week for 6 weeks. (**A**) Selected ventral view images from one representative mouse are shown. (**B**) Bioluminescence from primary tumors was quantified by region of interest (ROI) analysis of images obtained on the indicated days after orthotopic tumor implantation. Increases in bioluminescence correlate directly with tumor growth rate. (*P < 0.05, **P < 0.01 *vs* control; *P < 0.05, **P < 0.01 *vs* MPA) (**C**) Bioluminescence from locoregional metastatic tumors was quantified by region of interest (ROI) analysis of images obtained on the indicated days after orthotopic tumor implantation. Increases in bioluminescence correlate directly with tumor growth rate. (**P < 0.01, ***P < 0.001 *vs* control; **P < 0.01, ***P < 0.001 *vs* MPA).

After development and characterizing the orthotopic model for EC, we sought to evaluate the contribution of RANK overexpression to the early steps of tumor dissemination and distant metastasis. To detect the dissemination of tumor in a sensitive and non-invasive manner, *in vivo* BLI was first performed at day 7. At this early time points, 23 of the mice had tumor growth and confined to the uterus (Figure 4). Given the clinical importance of high RANK expression in EC, we focused on targeting RANK in a novel orthotopic model by using MPA. Selected ventral views from one representative mouse each group over time are shown in Figure 4A. Compared with control group, RANKL alone treatment showed a significant effect toward EC growth and pelvic metastases. Combination therapy reduced markedly the number of tumor nodules and the metastatic spread. Primary tumors and distant metastasis growth rate from the ventral region was quantified over time by means of BLI intensity in each group (Figure 4B–4C). Nonlinear regression plots were used to describe the relationship between BLI intensity and time after orthotopic implantation in RANKL-treated group (Supplementary Figure S3).

### MPA suppresses metastasis induced by RANK/RANKL via inhibiting AKT/β-catenin/Snail pathway in an orthotopic mouse model

**Figure 5:**
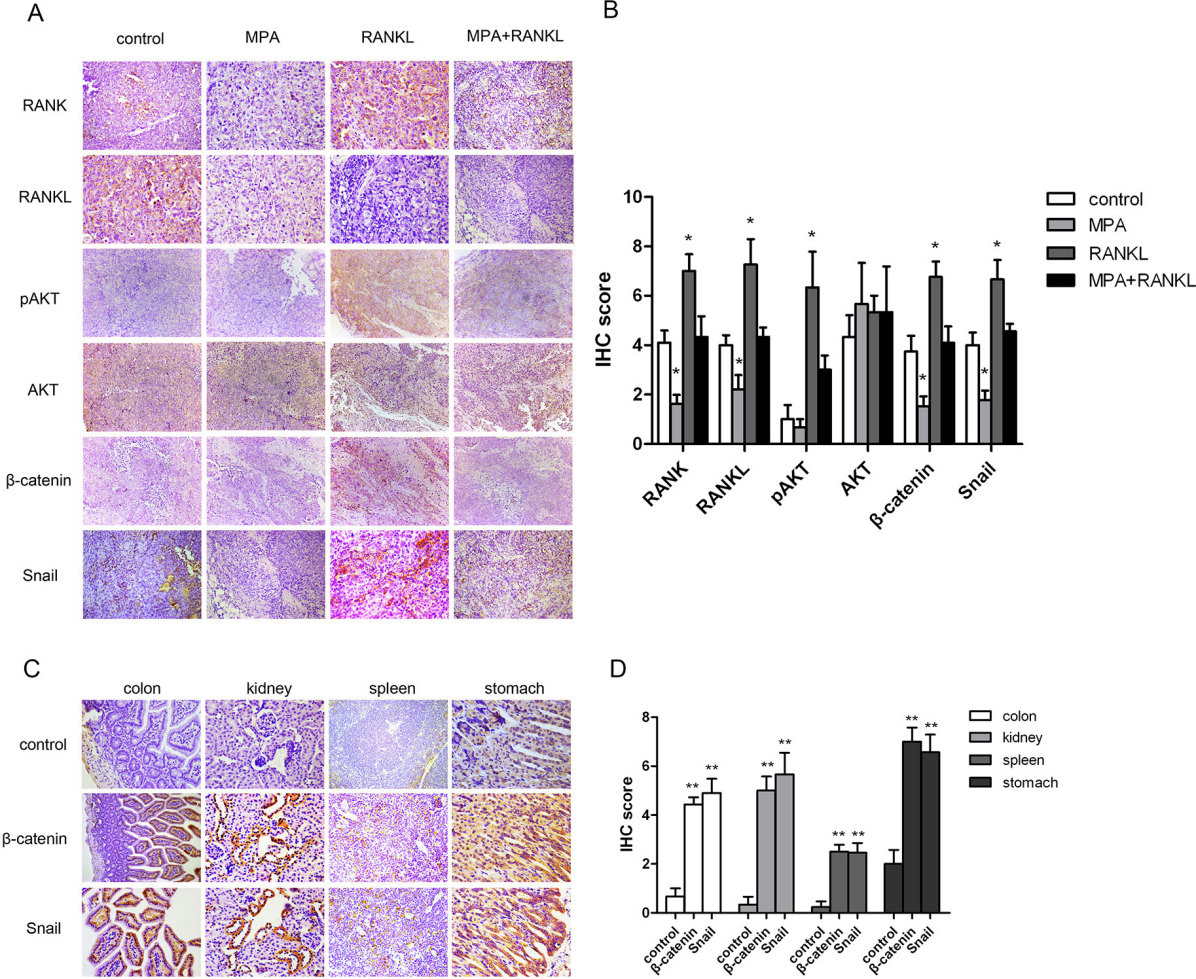
Expression of RANK/RANKL and metastasis-related proteins in mice samples (**A–B**) IHC analysis of RANK, RANKL, pAKT, AKT, β-catenin, and Snail in mice samples (left panel). Statistic summary of the IHC score were showed (right panel). (**C–D**) IHC analysis of β-catenin and Snail in the micrometastases of orthotopic xenograft model (left panel). Statistic summary of the IHC score were showed (right panel). Data were shown as the mean ± SEM (*P < 0.05,**P < 0.01 *vs* control).

A critical question to be considered was whether RANK levels associate with metastasis and expression of the pro-metastatic factors, as predicted by our hypothesis. To address this issue, we performed immunohistochemistry (IHC) staining to assess levels of these proteins in tumor specimen from mice. Based on the clinical significance of high RANK expression in EC, we focused on the orthotopic grafts of Ishikawa-Luc-Rank endometrial tumor-bearing mice after RANKL treatment. IHC analysis showed that the expressions of RANK, RANKL, pAKT, β-catenin and Snail were more intense than those found in the control group in the primary tumors. More importantly, the combination treatment showed a significant inhibitory effect toward pro-metastatic factors, indicating MPA suppressing metastasis induced by RANK/RANKL via inactivation of AKT/β-catenin/Snail pathway (Figure 5A). To account both for the stain intensity and the extent of staining, an IHC score was evaluated. Statistical summary of the IHC score were showed in Figure 5B. Additionally, tumors originating from the RANK overexpression Ishikawa-Luc-Rank cells developed micrometastases to distant colon, kidney, spleen, and stomach. In metastatic tumors, the both expressions of β-catenin and Snail were more intense compare with controls (Ishikawa-Luc endometrial tumor-bearing mice). The results were consistent with the statistical data (Figure 5C–5D).

### Verification of the RANK/RANKL effect on the AKT/β-catenin/Snail pathway in EC tissue specimens

**Figure 6:**
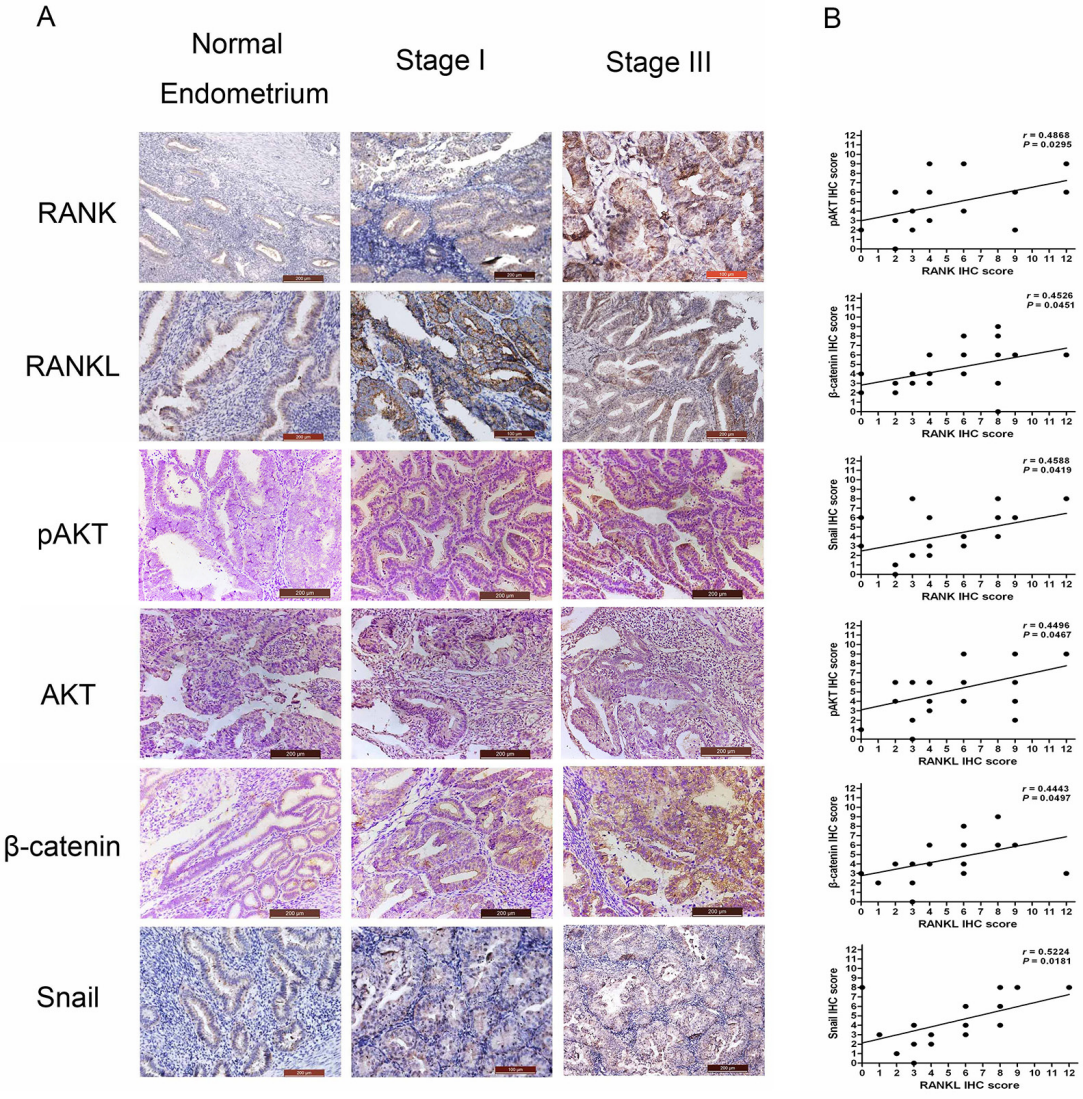
Expression of RANK/RANKL and prometastis-mediated factors in EC specimens (**A**) IHC detect the expressions of RANK, RANKL, pAKT, AKT, β-catenin, and Snail in normal endometrium and EC (stage I or III). (**B**) Expression correlations between RANK/RANKL and pAKT, β-catenin, and Snail, respectively. Statistical analyses were performed using Spearman's correlation coefficient test.

To verify the RANK effect on pro-metastatic proteins in EC tissue, we performed a semi-quantitative analysis of IHC staining in 20 specimens from 12 patients with EC. That stands for six specimens from each stage of EC (I/III) and ten specimens are served as the controls. IHC analysis confirmed that the levels of RANK, RANKL, pAKT, β-catenin, and Snail were significant higher in EC tissues (Figure 6A). Furthermore, RANK levels positively correlated with pAKT, β-catenin, and Snail, respectively (*P* = 0.0295, *P* = 0.0451, *P* = 0.0419). RANKL levels were also positively correlated with pAKT, β-catenin, and Snail, respectively (*P* = 0.0467, *P* = 0.0497, *P* = 0.0181) (Figure 6B).

Taken together, all of this data showing the metastatic characteristics with high level of RANK, and RANK/RANKL promotes EC metastasis involving AKT/β-catenin/Snail signaling pathway.

## DISCUSSION

Metastasis is widely recognized as intricate, multistep processes and closely associated with worse prognosis in tumors. It must be mentioned that the poor prognosis of patients with endometrioid EC has been associated with the extra-pelvic spread of the tumor [25]. Although surgery is the standard treatment for EC, identifying biomarkers that indicate the metastatic potential and outcome might help optimize treatment strategies. Several lines of evidence suggest that RANKL, where, in different contexts, acts as a dominant oncogene, a tumor promoter or a mediator of metastasis in certain tumor types. Thus, we focused on the effect of RANK/RANKL on EC metastasis. Specifically, we found that high RANK level in primary tumors is predictive of poor prognosis in patients with EC. Therefore, RANK may be a potential marker for EC metastasis and clinical prognosis.

The abnormal activation of the AKT pathway has been confirmed by experimental studies as a pivotal step toward the initiation and maintenance of human various tumors [26, 27]. The activation of AKT pathway triggers a cascade of biological events, which drives tumor progression. It has been reported not only to regulate β-catenin expression and activity but also phosphorylate the downstream IKKα to increase Snail expression [28, 29]. Moreover, β-catenin can interplay with Snail and other metastasis-related factors, for example, Hif-1α, Forkhead box 3a, and Forkhead box 4, suggesting that β-catenin complexes play vital roles in tumor dissemination and metastasis [30, 31]. The Snail transcription factor, which subsequently downregulates cadherins, has been demonstrated to induce the progression of distant metastasis *in vitro* cell models for breast, ovarian, and human prostate cancer [32–34]. Consistent with these findings, we found that AKT pathway is activated in Ishikawa-Luc-Rank cells compared with Ishikawa cells. We also showed that β-catenin and Snail are dramatically upregulated in Ishikawa-Luc-Rank cells and that AKT signaling inhibitor induces β-catenin and Snail suppression at protein and mRNA levels. Osteoprotegerin (OPG), a soluble decoy receptor, is capable of binding to RANKL and blocking its association with RANK. In this study, OPG, which blocks the RANK and RANKL interaction, dramatically inhibits the activity of AKT/β-catenin/Snail pathway.

In the current study, IHC analysis confirmed that the Ishikawa-Luc-Rank tumor molecular profile was representative of well-differentiated tumor (hormone receptors positive and low level of Ki-67), and the primary tumors resembled metastatic tumors with respect to the molecular pattern. The increased levels of pAKT, β-catenin and Snail in mice samples support the idea that RANK/RANKL promotes distant metastases in human EC orthotopic xenograft model. We have also verified of the RANK/RANKL effect on the AKT/β-catenin/Snail pathway in EC tissue specimens. Moreover, we detected a positive correlation between RANK and pAKT, β-catenin and Snail levels, as were RANKL. These results are consistent with our findings in EC cell lines. Finally, we demonstrated that the regulation of tumor cell metastasis and invasion by RANKL might be mediated partly through AKT/β-catenin/Snail-regulated factors. Proteasome inhibition is associated with the stabilization of E-cadherin and the suppression of Snail [35, 36]. Therefore, further molecular mechanisms involving proteasome activity are necessary to clarify the abnormal degradation of epithelial proteins and enhanced expression of mesenchymal proteins.

**Figure 7:**
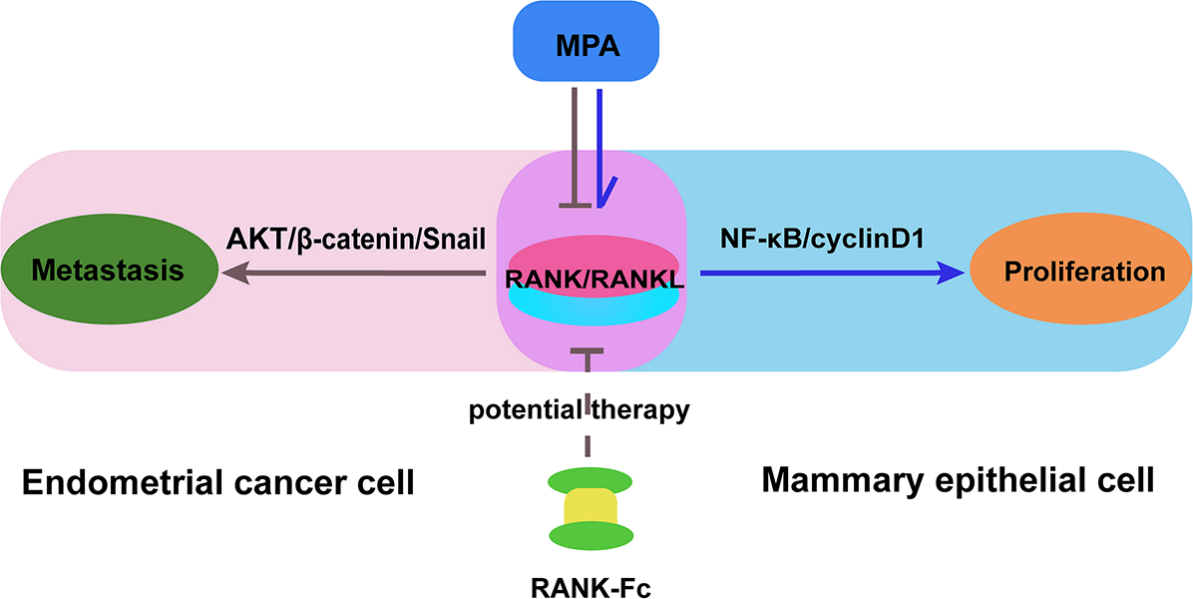
A proposed model for the effects of MPA and RANK-Fc on suppressing RANK/RANKL signal in different organs Combination of MPA and RANK-Fc suppresses distant metastasis induced by RANK/RANKL in EC via inactivation AKT/β-catenin/Snail signaling pathway, which suppress cell migration, invasion, and metastasis. Simultaneously, RANK-Fc weakens the proliferative effect of MPA on mammary gland.

Progesterone, especially synthesized derivatives such as MPA, is commonly used in hormone replacement therapy in postmenopausal women but markedly increase the risk of developing breast cancer. Dougall et al. demonstrated that MPA triggers the induction of RANKL in mammary-gland epithelial cells, and RANKL inactivation could prevent MPA-induced epithelial proliferation in progestin-driven breast cancer [20]. These data implicated that the RANK/RANKL is an important molecular link between progestin and epithelial carcinogenesis. And then, RANKL inhibition may be a potential approach to the management of breast cancer. On the contrast, we previously demonstrated that MPA efficiently inhibited the tumorigenicity in EC. Here, we also discovered that MPA could partially inhibit the distant metastasis induced by RANK/RANKL in EC. Considering the opposite effect of MPA on epithelial proliferation in EC and breast cancer, we proposed that MPA may have contrary effect on RANKL expression and activation in these two target organs. It takes the probability that RANKL may be a potential therapy target that both enhance the treatment effect of MPA in EC and avoid the stimulative effect on mammary gland (Figure 7). And our results also showed that OPG, a blockade of RANK and RANKL interaction, could significantly down-regulate the AKT/β-catenin/Snail pathway activation, which would proceed to suppress metastasis and invasion of EC. Further study of RANK-Fc (RANKL inhibitor) effect on EC may explain the different reaction of EC and breast cancer to MPA treatment.

In conclusion, our data suggest that MPA suppresses distant metastasis induced by RANK/RANKL in EC via inhibiting AKT/β-catenin/Snail pathway. RANK might be a biomarker for EC prognosis and a target for therapeutic intervention. Further investigations are warranted to explore the combination of MPA and RANK-Fc as a promising candidate for EC therapy, which could both inhibit the RANK/RANKL-induced metastasis in EC and weaken the proliferative effect of MPA on mammary gland.

## MATERIALS AND METHODS

### Cell cultures, constructs and stable transfection

Human EC cell line (Ishikawa) was purchased from the American Type Culture Collection (ATCC). Ishikawa cells were grown in Dulbecco's modified Eagle's medium-F12 (Gibco, Auckland, NZ) supplemented with 10% fetal bovine serum (Gibco, Carlsbad, CA) in a 5% CO_2_ humidified incubator at 37°C. Plasmid construction consisted of the firefly luciferase cDNA and human RANK. Human RANK was inserted at the BamHI/NheI position of the pL-TO-IRES-LUC vector. Then, Ishikawa cells were transfected with the newly constructed vector (pL-TO-IRES-LUC-rank) or a negative control vector (NC, pL-TO-IRES-LUC).

### Western blotting analysis

Total protein was extracted with lysis buffer (Beyotime, China) containing 1% dilution of the protease inhibitor PMSF. Equal amounts of protein were loaded into each lane of an SDS-PAGE gel, and transferred to PVDF membranes. After blocking the membrane with 5% skimmed milk at room temperature for 2 h and then incubated with primary antibodies against RANK (1:500), AKT (1:1000), p-AKT (1:1000), β-catenin (1:1000), Snail (1:500), and β-actin (1:1000) at 4°C overnight. Proxidase-linked secondary anti-rabbit (1:2500) and anti-mouse (1:2500) were used to detect bound primary antibodies.

### RNA extraction and PCR assays

Total RNA was extracted from culture cells using Trizol Reagent (Invitrogen). 1 μg of the total RNA was reverse transcribed into complementary DNA with the one-step Prime Script RT reagent kit (TaKaRa, China). Polymerase chain reaction was performed using forward primer RANK: 5′-AGCATTATGAGCATCTGGGAC-GG-3′ and reverse primer RANK: 5′-CAGCAAGCATT TATCTTCTTCATTCC-3′. PCR detection was normalized with β-actin, forward primer β-actin: 5′-CAGCCATGTA CGTTGCTATCCAGG-3′ and reverse primer β-actin: 5′-AGGTCCAGACGCAGGATGGCATG-3′.

### Immunohistochemistry (IHC) and assessments

Paraffin-embedded tissue specimens were dewaxed with xylene and then rehydrated using a graded alcohol series. The specimens were then incubated in 1:50 ethylene diamine tetraacetic acid for antigen retrieval for 20 min, and then 3% hydrogen peroxide for 10 min. Tissues were incubated with polyclone anti-RANK (1:250), anti-AKT (1:250), anti-pAKT (1:200), anti-β-catenin (1:200) and anti-Snail (1:200). The percentage of positive staining was graded as follows: 0 (0%), 1 (1% – 25%), 2 (26% – 50%), 3 (51% – 75%), or 4 (> 75%). Staining intensity was graded as 0 (negative), 1 (weak), 2 (medium), or 3 (strong). A final score was calculated by multiplying above scores, figuring out an immunoreactivity score of 0 – 12.

### Animals and tumor implantation procedure

Female athymic nude mice of 4 weeks of age were obtained from the Shanghai Experimental Animal Center of the Chinese Academy of Science. They were cared for in strict accordance with the recommendations in the guidelines set by the Care and Use of Laboratory Animal of China and the protocols of the Ethics Committee for Animal Experimentation of our institution. The animals were housed in individually ventilated cage units and were maintained under pathogen-free conditions. Food and water were provided *ad libitum*. Subcutaneous xenografts were established by injection of 1 × 10^7^ Ishikawa-Luc and Ishikawa-Luc-Rank tumor cells in 200 μl PBS into the flank of 12 nude mice (for each cell line). When the tumor had formed approximately 10 days after injection, the mice were sacrificed for necropsy by cervical dislocation after sedation.

For orthotopic implantation, 24 nude mice were anesthetized with 4% chloralhydrate i.p. (200 μl). An about 50 mm incision in length was made in the left lower flank to optimize exposure to the left uterus. One tissue sample of approximately 1 – 1.5 mm^3^ was implanted into the uterus and fixed with a 6 – 0 surgical suture to isolate the tissue block from the rest of the organs.

### *In vivo* bioluminescence imaging

Before imaging, mice were injected i.p. with D-Luciferin substract at 15 mg/ml in PBS at a dose of 150 mg/kg. The first imaging of orthotopic model was carried out 7 days after surgical implantation. For longitudinal assessment, BLI was conducted weekly up to six weeks to evaluate the orthotopic tumor growth and metastatic dissemination. The light emitted from the luciferase-tagged tumor cells was detected by IVIS^®^ Spectrum (Xenogen Corporation), digitalized and automatically displayed as a pseudo color overlay onto a gray scale animal image.

### Antitumorigenic effect of MPA *in vivo*

Seven days after the orthotopic implantation, the mice of each type were randomized into four groups: (a) vehicle, 200 μl PBS (control); (b) MPA (100 mg/kg body weight in 200 μl PBS); (c) RANKL (250 μg/kg bodyweight in 200 μl PBS); (d) MPA and RANKL. The MPA was injected three times weekly and RANKL i.p. twice weekly.

### Clinicopathologic analyses

All patients underwent surgical exploration and primary surgical staging as the initial treatment. According to the International Federation of Gynecology and Obstetrics stage (2009) [37], patients were classified into two groups: low stage (stage I and II) and high stage (III and IV). Disease-specific survival (DSS) was defined as the time from treatment completion until the date of death or the date of last contact. 5-year overall survival (OS) and 5-year progression-free survival (PFS) were evaluated by the Kaplan-Meier survival curves for RANK.

### Statistical analyses

Nonlinear regression plots were applied to describe the relationship between BLI intensity and time after orthotopic implantation. R^2^ values were used to evaluate the quality of the nonlinear regression model. Patients who were alive at the last follow-up or died from causes except for EC were considered as censored at the date of last follow-up. OS and PFS were estimated using the Kaplan-Meier product limit method. A two-sided log-rank test was used to test the differences between survival curves. DSS was estimated using univariate Cox proportional hazards regression. The spearman's correlation coefficient test was used for correlation detection. Continuous variables were assessed by unpaired Student's *T*-test or one-way ANOVA for multiple comparisons. A *P*-value of < 0.05 was considered statistically significant on two-tailed testing.

## SUPPLEMENTARY MATERIALS AND FIGURES


