# Supplementary material for: Clinical prognostic significance and pro-metastatic activity of RANK/RANKL via the AKT pathway in endometrial cancer
Source: Oncotarget. 2015 Dec 29;7(5):5564–75. doi: 10.18632/oncotarget.6795 (PMC4868706; doi:10.18632/oncotarget.6795)
Supplement: Supplementary file 1 [file oncotarget-07-5564-s001.pdf]

Clinical prognostic significance and pro-metastatic activity of RANK/RANKL via the AKT pathway in endometrial cancer

Supplementary Materials

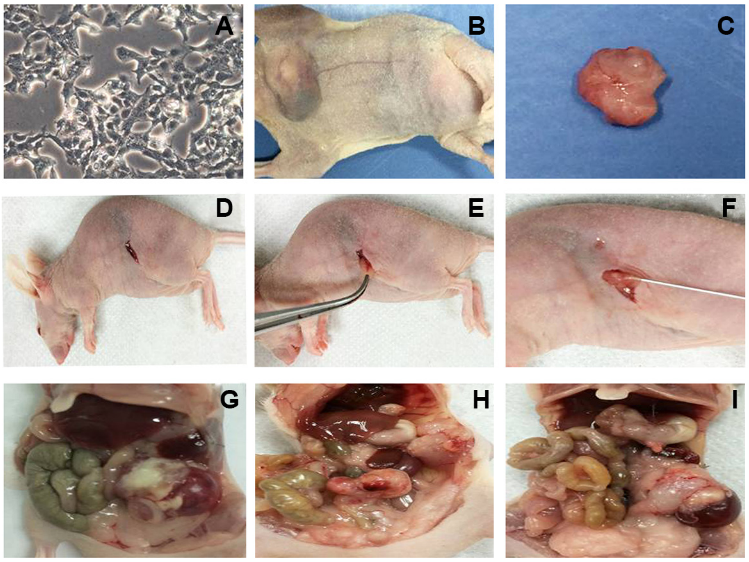

Supplementary Figure S1: Development and characterization of an orthotopic endometrial cancer model.

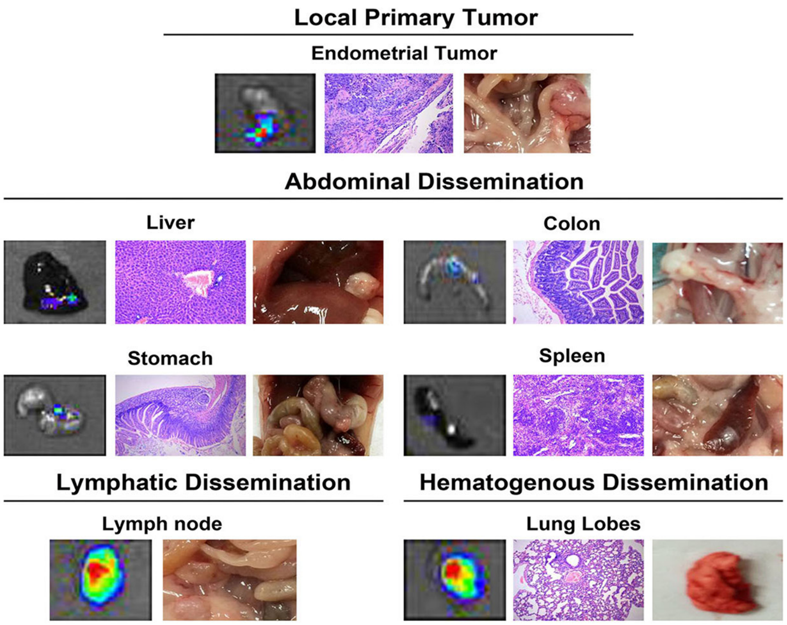

Supplementary Figure S2: *Ex vivo* bioluminescence images, H & E staining and necropsy images of the orthotopic Ishikawa-Luc-Rank endometrial tumor-bearing mice.

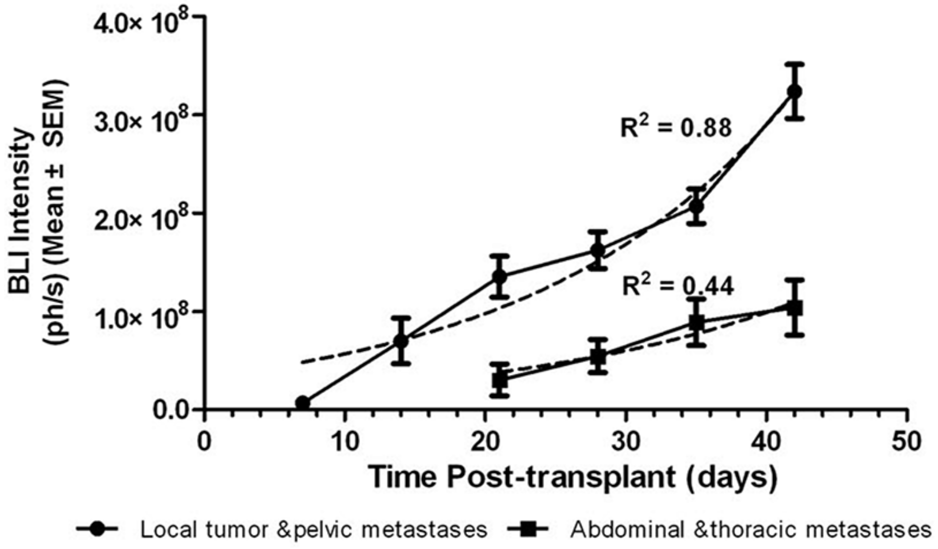

Supplementary Figure S3: Nonlinear regression plots were used to describe the relationship between BLI intensity and time after orthotopic implantation in RANKL-treated group.
